# Intact cell MALDI-TOF mass spectrometric analysis of *Chroococcidiopsis* cyanobacteria for classification purposes and identification of possible marker proteins

**DOI:** 10.1371/journal.pone.0208275

**Published:** 2018-11-29

**Authors:** Marek Šebela, Eva Jahodářová, Martin Raus, René Lenobel, Petr Hašler

**Affiliations:** 1 Department of Protein Biochemistry and Proteomics, Centre of the Region Haná for Biotechnological and Agricultural Research, Faculty of Science, Palacký University, Olomouc, Czech Republic; 2 Department of Botany, Faculty of Science, Palacký University, Olomouc, Czech Republic; Universita degli Studi di Parma, ITALY

## Abstract

Cyanobacteria represent a bacterial phyllum characteristic by the ability to photosynthesize. They are potentially applicable for the production of useful compounds but may also cause poisoning or at least health problems as they can produce cyanotoxins. The introduction of a fast methodology is important not only for fundamental taxonomic purposes, but also for reliable identifications in biological studies. In this work, we have used matrix-assisted laser desorption/ionization time-of-flight mass spectrometry of intact cells to study *Chroococcidiopsis* strains. A library of the obtained reference mass spectra containing characteristic peptide/protein profiles was examined by software tools to characterize similarities and differences applicable for diagnostics and taxonomy. Both a similarity tree and heat map constructed from the mass spectrometric data proved consistent with 16S rRNA sequencing results. We show as novelty that a binary matrix combining ferulic and sinapinic acids performs well in acquiring reproducible mass spectra of cyanobacteria. Using the matrix solvent, a protein extraction from cells was done. After polyacrylamide gel electrophoresis, the separated protein fractions were in-gel digested and the resulting peptides analyzed by liquid chromatography coupled with tandem mass spectrometry. For the first time, photosystem protein components, phycobilisome proteins, electron transport proteins, nitrogen-metabolism and nucleic acids binding-proteins, cytochromes plus other enzymes and various uncharacterized proteins could be assigned to characteristic peaks in the mass spectrometric profiles and some of them suggested as markers in addition to 30S and 50S ribosomal proteins known from previous studies employing intact cell mass spectrometry of microorganisms.

## Introduction

Cyanobacteria belong to the oldest continuously living organisms on earth. First they appeared approximately 3.5 billion years ago [1]. At that time, their photoautotrophic metabolism allowed dominance. During the whole life history, they gradually adapted themselves to numerous habitats from tropical to arctic areas including both aquatic and terrestrial environments. Despite their simple prokaryotic cell structure, cyanobacteria exhibit a high morphological variability, which represented the main criterion to build up classification schemes for a long time. Geitler introduced a sophisticated botanical classification system [2], which was kept in use almost for the whole 20th century. However, due to the increasing knowledge of biodiversity, cyanobacteria have been included into the bacteriological taxonomy which pays a big attention to cellular traits characterized by microbiological methods [3].

In the 1980s, a revolutionary taxonomy combining both the Geitlerian and bacteriological system was introduced [4]. The current status largely combines the above-mentioned botanical classification, knowledge on ecology of species and results of molecular analysis of 16S rRNA genes and ITS (internal transcribed spacer) regions in DNA. A revision of the established cyanobacterial system, which strongly emphasizes the molecular analysis, has been suggested by Komárek et al. [5]. Yet, despite the recent progress, many questions of interspecific and intraspecific diversity have remained unanswered. There are numerous genera and species with an unclear taxonomy because of their high level of polyphyletism. For example, members of the genus *Synechococcus* possess a very similar morphology including single cells, often elongated with an asymmetrical cell division, and a few celled pseudofilamentous formations, which differ in their 16S rRNA gene/ITS region [6]. As it may be useful in many human activities (e.g. in applied phycology, hydrobiology or environmental studies), it is important to ensure correct identification of cyanobacterial species. The current trend heads toward a rapid identification using instrumental methods replacing traditional microscopic investigations. However, 16S rRNA/ITS analyses are time consuming (a few days are needed including DNA sequencing), which limits this approach as a possible routine. Therefore, modern alternatives should offer a solution, which is fast, accurate, sensitive, robust and cheap.

Matrix-assisted laser desorption/ionization time-of-flight mass spectrometry (MALDI-TOF MS) has recently been introduced into a routine use in laboratories to characterize and type microbes and becomes promising to replace the established biochemical or molecular biological techniques. It allows fast identifications of various bacteria, fungi (including yeasts) or protozoa and also parasitic insects such as ticks or fleas [7–10]. A standard approach for microorganisms resides in using intact cells (i.e. without any destructive pretreatment) or peptide/protein extracts. Then peptide/protein profile mass spectra are acquired, which is facilitated by choosing a proper matrix compound as a part of the sample preparation step [11]. The mass range for the acquisition is typically between *m/z* 1000 and 20,000 and the profile spectrum pattern is finally compared with a reference spectral database for identification [12].

MALDI-TOF MS in identification of cyanobacteria has not advanced forward yet compared to its application for human pathogenic bacteria and fungi. After first attempts to introduce MALDI-based approaches for analyzing oligopeptide secondary metabolites of *Microcystis* and *Anabaena* strains [13,14], other important papers dealing with cyanobacteria (and demonstrating the usefulness of the methodology for both identification and rapid classification of strains) appeared only very recently [15,16]. Thus there is still a knowledge gap as regards to the optimization of the sample preparation procedure, addressing the issues of reproducibility and data interpretation strategies. In this study we have attempted to develop an efficient, reliable and easy-to-use MALDI-based procedure for *Chroococcidiopsis* strains and demonstrated the applicability of two software solutions for data evaluation.

## Materials and methods

### Biological material and microscopy

Inocula to grow cultures were obtained from CCALA (Culture Collection of Autotrophic Organisms, Institute of Botany of the AS CR, Třeboň, Czech Republic), UPOC (Collection of phytopathogenic microorganisms in the Department of Botany, Faculty of Science, Palacký University in Olomouc, Czech Republic), CAUP (Culture Collection of Algae of Charles University in Prague) and PCC (the Pasteur Culture Collection of Cyanobacteria, Institut Pasteur, Paris, France); see Table 1. The analyzed strains were incubated in sterile liquid Z medium [17] in 100-ml Erlenmeyer flasks or on 1.5% agar plates (9 cm in diameter) for three weeks (temperature: 22°C; 16/8h light/dark cycle; illumination of 20 μmol·m^-2^·s^-1^). Cells were harvested by centrifugation at 260 *g* for 10 min (liquid cultures) or simple scraping (from agar plates). Microscopic inspections were performed using a light microscope Zeiss AxioImager (Zeiss D512 camera, objective Zeiss Plan-Apochromat 100× / N.A. 1.46, Oil, DIC). Microphotographs were processed using AxioVision 4.9.1. Package (Zeiss); see in S1 Fig.

**Table 1:** A group of cyanobacteria samples analyzed in this work.

No.	Species	Strain	Year	Medium	Place of origin	Habitat
1	*Chroococcidiopsis cubana*	CCALA 040	1964	Liquid	San Diego, Cuba	mineral spring, periphyton
2	*Chroococcidiopsis cubana*	CCALA 041	1965	Agar	Habana, Cuba	basin
3	*Chroococcidiopsis cubana*	CCALA 042	1965	Agar	Santa Fe, Cuba	mineral spring, stone
4	*Chroococcidiopsis cubana*	CCALA 043	1965	Liquid	Santa Fe, Cuba	mineral spring, stone
5	*Chroococcidiopsis cubana*	CCALA 044	1965	Liquid	Nueva Gerona, Cuba	ditch
6	*Chroococcidiopsis* cf. *cubana*	CCALA 045	1966	Liquid	Pinar del Rio, Cuba	pool
7	*Chroococcidiopsis* cf. *cubana*	CCALA 047	1968	Agar	Horní Benešov, Czech Republic	reservoir, periphyton
8	*Chroococcidiopsis cubana*	UPOC 17/2013	2013	Agar	Jacksonville, USA	aerophytic
9	*Chroococcidiopsis cubana*	UPOC 18/2013	2013	Agar	Jacksonville, USA	aerophytic
10	*Chroococcidiopsis cubana*	UPOC 19/2013	2013	Agar	Jacksonville, USA	aerophytic
11	*Chroococcidiopsis cubana*	UPOC 20/2013	2013	Agar	Jacksonville, USA	aerophytic
12	*Chroococcidiopsis cubana*	UPOC 34/2013	2013	Agar	Jacksonville, USA	aerophytic
13	*Chroococcidiopsis cubana*	UPOC 115/2013	2013	Agar	Jacksonville, USA	aerophytic
14	*Chroococcidiopsis cubana*	UPOC 1UNF/2013	2013	Agar	Jacksonville, USA	aerophytic
15	*Chroococcidiopsis epilithica*	UPOC 164/2015	2016	Agar	Jacksonville, USA	aerophytic
16	*Chroococcidiopsis* sp.	CCALA 046	1968	Agar	Constanta, Mamaia, Romania	epipsamic
17	*Chroococcidiopsis* sp.	CCALA 051	1962	Liquid	Belianské Tatry, Slovakia	no data available
18	*Chroococcidiopsis* sp.	CCALA 052	1962	Liquid	Yerevan, Armenia	stone, subaerophyte
19	*Chroococcidiopsis* sp.	UPOC 169/2016	2016	Liquid	Vietnam	aerophytic
20	*Chroococcidiopsis thermalis* (?)	CCALA 048	1964	Agar	Cuba	no data available
21	*Chroococcidiopsis thermalis*	CCALA 050	1975	Agar	Piešťany, Slovakia	thermal spring, periphyton
22	*Neosynechococcus sphagnicola*	sy1	2010	Liquid	Námestovo, peat bog Klin, Slovakia	endophytic
23	*Synechococcus* sp.	UPOC S3	2006	Liquid	Loštice, Czech Republic	endozooic
24	*Synechococcus* sp.	UPOC S4	2006	Liquid	Loštice, Czech Republic	endozooic
25	*Synechococcus* sp.	UPOC 71b/2013	2012	Liquid	Jacksonville, USA	epipelic
26	*Gloeobacter violaceus*	PCC 7421	1972	Liquid	Vierwaldstättersee, Switzerland	aerophytic

### Chemicals

Ferulic acid (FA) and sinapinic acid (SA) with a declared MALDI matrix quality were purchased from Sigma-Aldrich Chemie (Steinheim, Germany) as well as trichloroacetic acid (TCA). Bacterial Test Standard, Peptide Calibration Standard II and α-cyano-4-hydroxycinnamic acid (CHCA) were from Bruker Daltonik (Bremen, Germany). Organic solvents (e.g. acetonitrile—ACN, acetone or isopropanol), formic acid (FoA) and trifluoroacetic acid (TFA) were from Merck (Darmstadt, Germany). All other chemicals were of analytical purity grade.

### Preparation of intact cells

Aliquots of the harvested cyanobacterial cells were picked up by a spatula and washed tree times by gentle vortexing with deionized water in a 1.5-ml tube. During the washing steps, the cells were collected by a short centrifugation at 5,000 × *g* for 5 min. Finally, the biomass was suspended in the same volume of deionized water. The exact concentration of cyanobacterial cells was not determined as it was not necessary–the aim was to develop a simple and fast procedure.

### Intact cell MALDI mass spectrometry (IC MALDI-MS)

MALDI-TOF MS of cyanobacterial cells was performed on a Microflex LRF20 instrument equipped with a nitrogen laser; wavelength: 337 nm, pulse repetition rate: 60 Hz (Bruker Daltonik). Mass spectra were acquired in the reflectron positive ion mode using an acceleration voltage (IS1) of 19.0 kV, extraction voltage (IS2) of 15.5 kV, lens voltage of 9.0 kV, reflectron voltage of 20.0 kV, detector voltage of 1668 V and pulsed ion extraction delay time of 500 ns. The examined mass region ranged from *m/z* 1000 to *m/z* 20,000 and the instrument was calibrated externally using the Bacterial Test Standard covering average masses between 3637.8 and 16952.3 Da (Bruker Daltonik). A binary matrix system was used for MALDI, which consisted of ferulic acid (5 mg·ml^-1^) and sinapinic acids (15 mg·ml^-1^) dissolved together in a mixture of ACN and 2% (v/v) TFA in a volume ratio of 7:3. The cell suspension was applied in an amount of 1 μl to the target plate (MSP BigAnchor 96 BC microScout Target; Bruker Daltonik), mixed with the same volume of the matrix solution and left to dry for crystallization of sample/matrix cocrystals. Alternatively, during the protocol development, the cell suspension sample deposited on the target was overlaid with 1 μl of 25% (v/v) FoA, air-dried, and again overlaid with 1 μl of matrix solution. The acquisition software was flexControl 3.4 and the spectral data were checked and saved using flexAnalysis 3.4 (all by Bruker Daltonik) without any smoothing or other processing steps. For each sample, MALDI mass spectra were acquired from at least ten independent spots and accumulated individually from 1000 laser shots for the construction of reference spectral profiles.

### Processing of data from IC MALDI-MS

The obtained data sets were processed using two software tools: MALDI Biotyper 3.1 (Bruker Daltonik) and Biospean, https://software.cr-hana.upol.cz/biospean [18]. In the Biotyper, reference spectra (main spectral projections i.e. MSPs) were first created from the experimental data and saved as a library. This library was then used for searches and to construct a taxonomy tree. For processing in the Biospean, the acquired mass spectra were first exported from flexAnalysis 3.4 (Bruker Daltonik) as TXT formatted files. Virtual reference spectra were made by including peaks represented in at least 70% of the acquired spectra with a tolerance of ±2 mass units for the *m/z* values of the peak maxima. These reference spectra were compared with each other to evaluate their similarity. The score value in Biospean is derived from the number of identical peak positions found divided by the total number of detected peaks in the inspected spectrum [18].

### Protein extraction

Cyanobacterial cultures grown in 500-ml Erlenmeyer flasks were transferred to 250-ml polypropylene centrifugal cuvettes and spun down at 13,000 × *g* for 30 min. The pellets in each cuvette were suspended with the help of a glass rod in 5 mL of the MALDI matrix solvent i.e. ACN/2% (v/v) TFA, 7:3, v/v. Then the suspensions (combined together for each species) were transferred to 15-ml tubes and shaken in a thermomixer (Eppendorf, Hamburg, Germany) at 23°C and 750 RPM for 14 h. Protein extracts were collected as supernatants by centrifuging at 10,000 × *g* for 20 min and stored frozen at -20°C. On freezing, two phases separated. The bottom phase was either colorless or faintly greenish ice; the upper phase was liquid and colored. Prior to further analysis, the upper liquid phase was removed by pipetting and discarded. On thawing, the bottom aqueous phase (5–6 ml) was first concentrated on a rotary vacuum evaporator at 55°C to a volume of around 2 ml and then dried out completely in a vacuum centrifuge at 45°C. The solid material was dissolved in 400 μl of the Laemmli sample buffer, pH 6.8, containing 5% β-mercaptoethanol [19] with the help of a short sonication and heating at 100°C for 5 min. The final solution was made alkaline by adding 50 μl of 2 M NaOH and, if not used immediately for electrophoresis, stored frozen at -20°C.

### Gel electrophoresis

Protein electrophoresis was conducted in 1-mm thick slab polyacrylamide minigels using a Mini-Protean II apparatus and a PowerPac Universal power supply (Bio-Rad, Hercules, CA, USA). The separating (10% T, 2.7% C) as well as stacking gel (4% T, 2.7% C) were both cast according to Laemmli [19]. Protein extract samples in the sample buffer (see above) were loaded in 25-μL aliquots and separated together with protein standards at a constant voltage of 130 V until the bromophenol blue tracking dye reached the bottom of the gel slab. The electrode buffer (pH 8.8) contained 0.025 M Tris-HCl, 0.192 M glycine and 0.1% (w/v) sodium dodecyl sulfate [19]. Proteins in gels were fixed in 10% (w/v) TCA for 10 min. After washing steps in 1% (w/v) TCA for at least 2 h and three times briefly in deionized water, they were stained by Bio-Safe Coomassie Stain solution (Bio-Rad).

### In-gel digestion

Prior to protein identification by mass spectrometry, in-gel digestions were performed according to a standard protocol with destaining, reduction and alkylation steps preceding the addition of trypsin [20]. A raffinose-modified thermostable trypsin was employed [21]. Finally, peptides were extracted from the digests using 5% (v/v) FoA/ACN, 1:2, v/v, as an extraction solvent and dried out in a vacuum centrifuge. They were stored until use at -20°C [20].

### Protein identification

Peptides from in-gel digests were first reconstituted in 12 μl of 0.1% (v/v) TFA with the aid of a short sonication. Then they were separated by nanoflow liquid chromatography (nanoLC) on a C18 reversed phase followed by electrospray ionization quadrupole time-of flight (ESI-Q-TOF) MS/MS or MALDI-TOF/TOF MS/MS analyses. The instrumental setup thus consisted of two mass spectrometers: a Q-TOF maXis and ultrafleXtreme MALDI-TOF/TOF (Bruker Daltonik), both coupled to a UltiMate 3000 RSLCnano liquid chromatograph (Thermo Fisher Scientific, Germering, Germany). In the latter case, offline coupling was via a Proteineer fc II liquid handler (Bruker Daltonik) for post column MALDI preparation of nanoLC separated peptide fractions; CHCA was used as a matrix according to manufacturer’s instructions.

For nanoLC-ESI-MS/MS, the loading solvent was 2% (v/v) FoA, mobile phase A was 2% (v/v) ACN containing 0.4% (v/v) FoA and mobile phase B was 90% (v/v) ACN containing 0.4% (v/v) FoA. The precolumn used (75 μm × 20 mm) ended with a frit (IntegraFrit, cat. no: IF360-75-50-N-5; New Objective Inc., Woburn, MA, USA) and was packed with 5 μm C18 sorbent (ReproSil-Pur 200, C18-AQ; Dr. Maisch GmbH, Ammerbuch-Entringen, Germany). The analytical column (75 μm × 200 mm) ended with a pointed emitter (PicoTip, cat. no: FS360-50-8-N-5-C25; New Objective Inc., Woburn, MA, USA) and was packed with 5 μm C18 sorbent (ReproSil-Pur 200, C18-AQ; Dr. Maisch GmbH). Both nano columns were prepared using a pressure vessel „Sample and Column Loader”(Proxeon Biosystems, Thermo Fisher Scientific, Waltham, MA, USA) pressurized with helium. For nanoLC-MALDI-MS/MS, the loading solvent was 2% (v/v) ACN containing 0.05% (v/v) TFA, mobile phase A was 0.05% (v/v) TFA and mobile phase B was 80% (v/v) ACN containing 0.05% (v/v) TFA. The precolumn used was a Nano Trap column (100 μm × 20 mm; nanoViper inlet/outlet) packed with Acclaim PepMap C18 silica particles (5 μm particle size, 100 Å pore size), the analytical column was Acclaim PepMap RSLC column (75 μm × 150 mm; nanoViper inlet/outlet) with C18 silica particles (2 μm particle size, 100 Å pore size), both by Thermo Fisher Scientific.

A detailed description of the gradient programming, instrumental settings, calibration and software control has been provided elsewhere [22]. MGF formatted data files were uploaded to ProteinScape 3.1 for MS/MS database searching against Swiss-Prot amino acid sequence database. Parameters of the searches have also been described [22].

### PCR amplification and sequencing

Genomic DNA was isolated from approximately 50 mg of fresh biomass using an UltraClean Microbial DNA Isolation Kit (MOBIO, Carlsbad, CA, USA) following the manufacturer’s manual. DNA quality and consistence was checked on a 1.5% agarose gel stained with GelRed (Biotinum Inc., Hayward, CA, USA). DNA was quantified using a NanoDrop 1000 (Thermo Fisher Scientific, Wilmington, DE, USA). PCR amplifications of 16S rRNA were achieved using the following primers: forward P2 (5‘-GGGGAATTTTCCGCAATGGG-3‘) and reverse P1 (5‘-CTCTGTGTGCCTAGGTATCC-3‘) as previously described [23]. The PCR reaction mixture, in a total volume of 40 μl, contained 17 μl of sterile water, 1 μl of each primer (0.01 mM concentration), 20 μl of FastStart PCR Master (Roche Diagnostics GmbH, Mannheim, Germany), and 1 μl of template DNA (50 ng·μl^-1^). The other conditions were the same as described in Dvořák et al. [24]. The same pair of primers as above was used for commercial sequencing. Additionally, two other internal primers were added: P5 (5‘-TGTACACACCGCCCGTC-3‘) and P8 (5’-AAGGAGGTGATCCAGCCACA-3‘) [23,25]. We used Sequencher 5.1 (Gene Codes Corporation, Ann Arbor, MI, USA) to assemble and proofread sequences.

### Phylogenetic analyses with DNA sequences

Multiple DNA sequence alignment was performed in MAFFT with the E-INS-i algorithm [26]. The corresponding tree was rooted to *Gloeobacter violaceus* as an outgroup. The most appropriate model (HKY+I) for Bayesian inference was determined in jModelTest 0.1.1 [27] based on both the Bayesian and Akaike information criteria. The 50% majority consensus tree was constructed in MrBayes 3.2.3 [28]. Two separate runs were performed, each with three heated and one cold chains for 100,000,000 generations. The sampling frequency was for each 5000th generation. Twenty five percent trees were discarded as burn-in. Maximum likelihood analysis was performed in RaxML 8.0.2 [29] with a GTR+G model using bootstrapping with 1000 replicates.

## Results

The studied cyanobacteria were strains of several species from the genus *Chroococcidiopsis* (*Chroococcidiopsis cubana*, *Chroococcidiopsis thermalis*, *Chroococcidiopsis* spp.). The experimental set was completed by taxonomically distant species such as *Gloebacter violaceus*, *Neosynechococcus sphagnicola* and several representatives of the genus *Synechococcus* to ensure outgroups for phylogenetic analyses. In total, there were 26 samples of cyanobacteria available (Table 1). Microscopic photographs of cells of the studied species are provided in S1 Fig. Upon harvesting, the cells were intact and further used in the form of a 1:1 (v/v) suspension in sterile water.

IC MALDI-MS with the harvested cyanobacterial cells was performed on Microflex LRF 20 MALDI-TOF mass spectrometer. It is a standard benchtop instrument applicable for routine identifications of microorganisms [30]. The measurements were carried out in the reflectron positive ion mode to increase mass resolution. In consequence, masses were measured as accurately as possible, which allowed more relevant comparisons with those deduced from the amino acid sequences of the identified proteins (see further). A binary FA:SA matrix was chosen to prepare MALDI sample spots on the target. This combination of matrix compounds has been shown a good choice to measure MALDI profile spectra of fungal intact cells or spores [11,31]. Evaluation tests with CHCA, saturated solution in 50% ACN with 2.5% (v/v) TFA (recommended for bacteria by the manufacturer of the MALDI Biotyper system), were not satisfactory enough as regards to the number and intensity of peaks.

**Fig 1:**
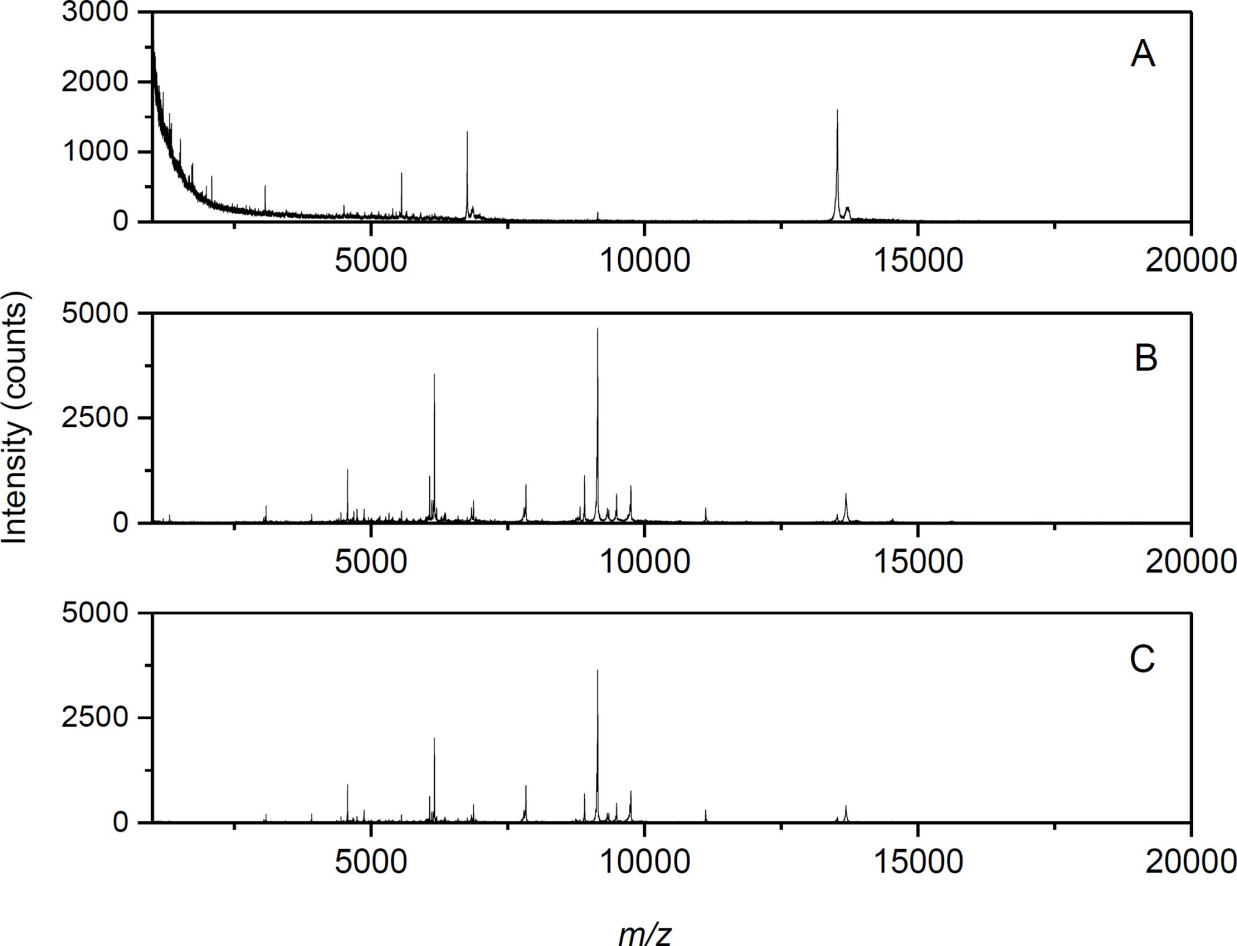
Intact cell MALDI-MS of cyanobacteria: the use of different TFA concentrations. The measurements were carried out on Microflex LRF20 MALDI-TOF instrument with *Chroococcidiopsis cubana* (UPOC 18/2013) cells and a binary matrix composed of FA and SA (5:15 mg·ml^-1^) dissolved in ACN/TFA, 7:3, v/v. The dried-droplet sample preparation technique was used and the TFA concentration prior to mixing with ACN was 0.1%, v/v (panel A), 2%, v/v (panel B) or 5%, v/v (panel C). As can be seen, choosing 2% TFA resulted in spectra of the highest quality.

**Fig 2:**
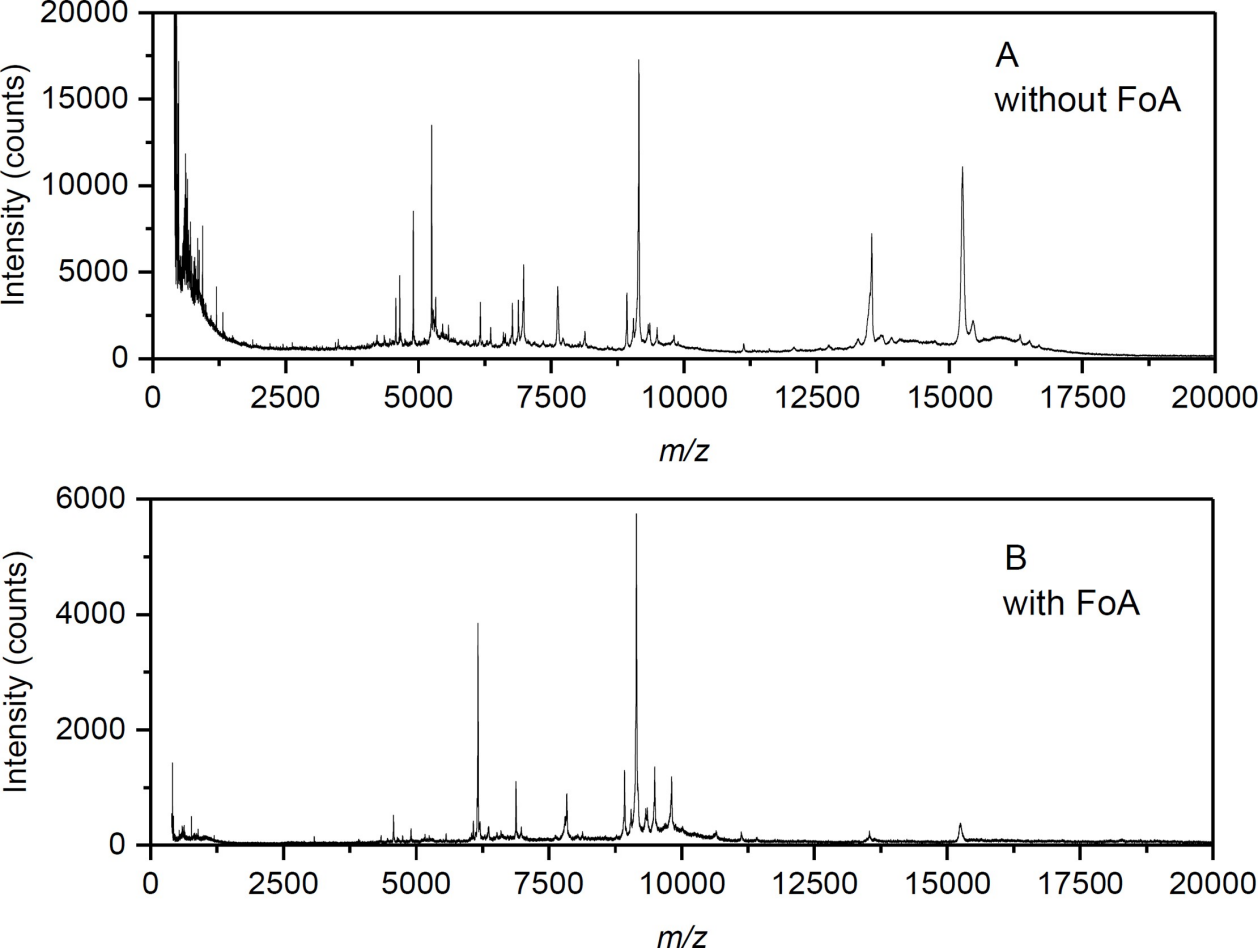
Intact cell MALDI-MS of cyanobacteria: evaluation of FoA application. The measurements were carried out on Microflex LRF20 MALDI-TOF instrument. Panel A shows a mass spectrum acquired after employing the dried-droplet sample preparation technique with the optimized binary matrix (FA:SA, 5:15 mg·ml^-1^ in ACN/2% v/v TFA, 7:3, v/v). Panel B shows a different result when cyanobacterial cells (*Chroococcidiopsis* spp., UPOC 169/2016) were first spotted on the target plate, overlaid with 25% (v/v) FoA, air-dried, and finally overlaid with the above matrix solution prior to spectrum acquisition.

Optimizations of the sample preparation procedure were necessary to transfer the arrangement developed originally with fungi to cyanobacteria. They included: 1) adjusting the concentrations of matrix and TFA, and 2) evaluation of the sample deposition technique. FA:SA mixtures, 10:30 mg·ml^-1^ and 5:15 mg·ml^-1^, in ACN with 0.1–5% (v/v) TFA, 7:3, v/v were tested. The latter was found better as regards to the number and intensity of peaks in the mass spectrum. Fig 1 demonstrates the differences in MALDI-TOF mass spectra when 0.1%, 2%, or 5% TFA was used in the matrix solution and the cells were processed by a dried droplet sample deposition technique (the suspension of cells was mixed with matrix directly on the target plate). As can be seen, the use of 2% TFA resulted in the best peptide/protein profiles. Besides the dried droplet, two other sample deposition techniques were examined: a mixed-volume, when the cell suspension was premixed in a ratio of 1:1 (v/v) with matrix solution and then applied to the target, and a two-layer technique (sample spots were overlaid with 25% FoA [32], air-dried, and then overlaid with the matrix solution). Anyway, the dried droplet technique was found optimal (Fig 2). A library of intact cell MALDI mass spectra with peptide/protein profiles of the studied cyanobacteria was then created. Examples of species pairs yielding similar or distant spectral profiles in a cursory comparison are shown in S2 Fig.

**Fig 3:**
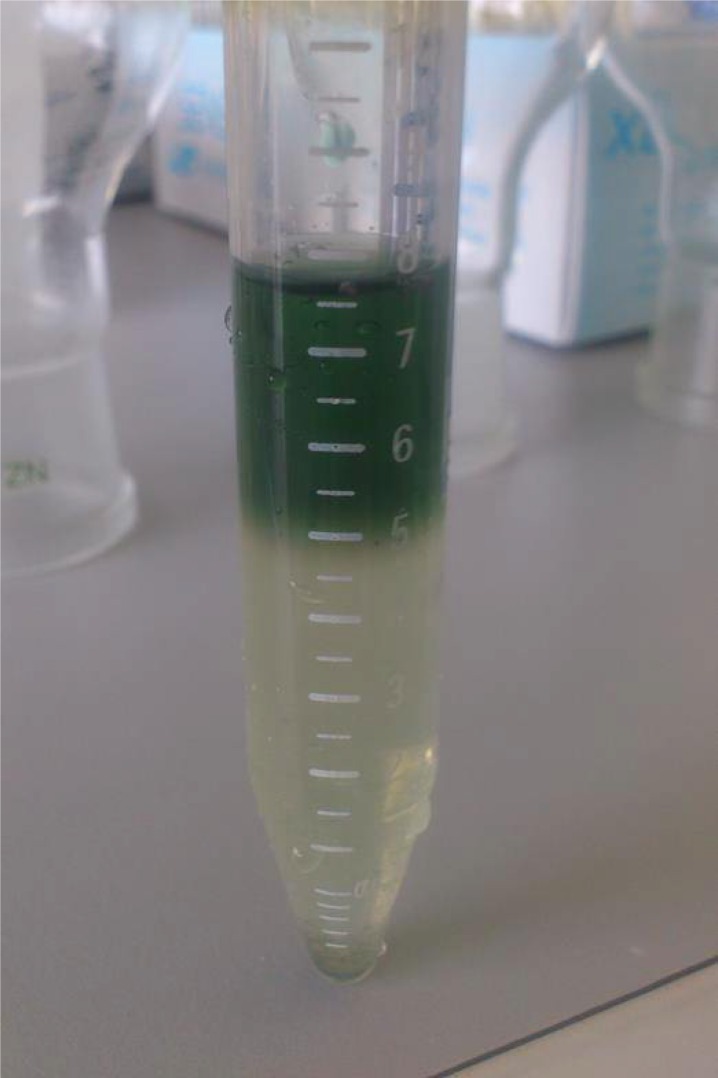
Protein extraction from harvested cyanobacterial cells. This figure shows a photograph of the extract obtained by treating *Synechococcus* sp.cells (UPOC S4) with ACN/2% (v/v) TFA, 7:3, v/v, after its storage at -20°C. On freezing, two phases separated. The upper phase was liquid, organic and colored because of the presence of photosynthetic pigments. The bottom phase was solid and almost colorless. It contained a vast majority of the extracted proteins and was further used in protein identification analyses.

**Fig 4:**
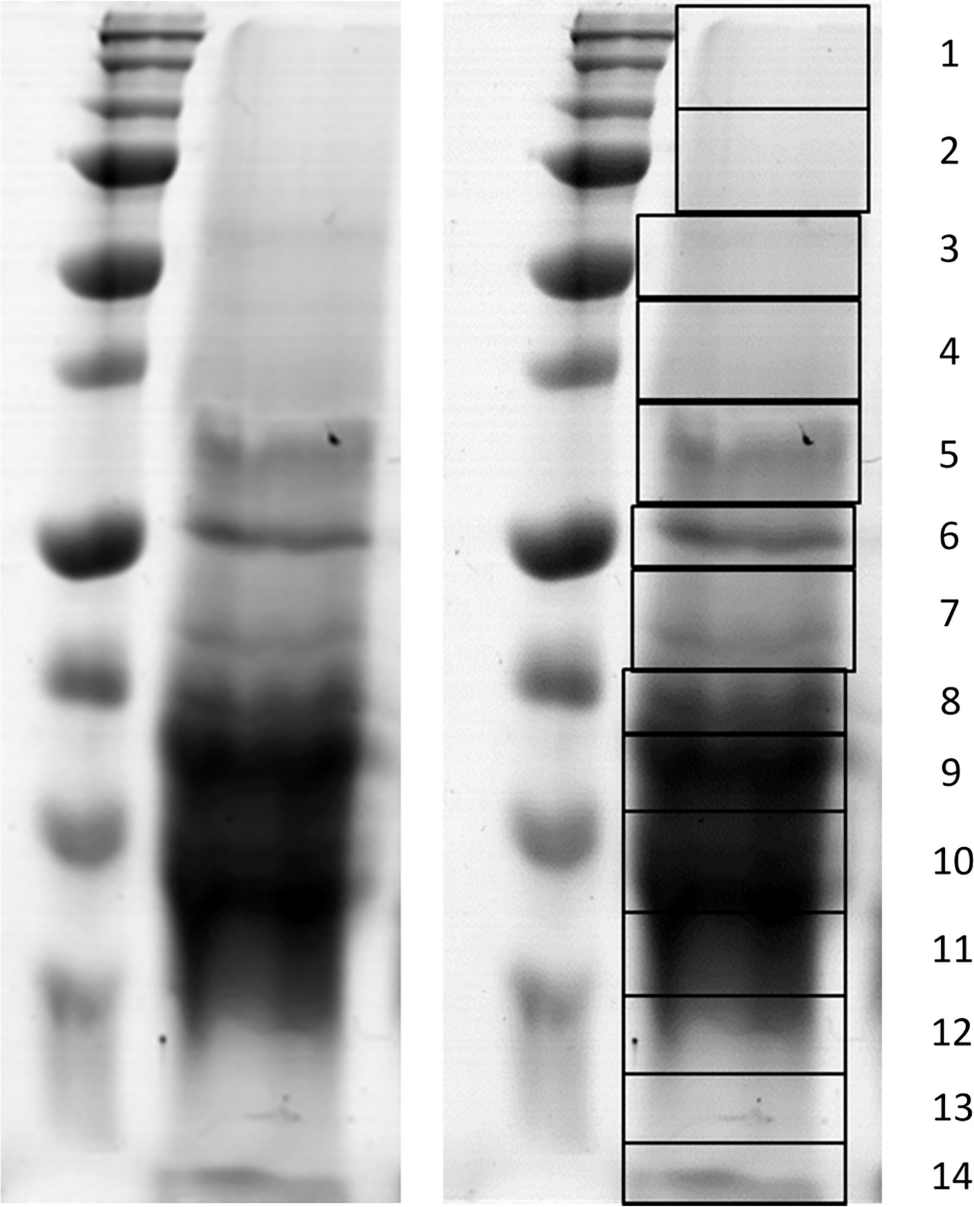
SDS-PAGE separation of extracted cyanobacterial proteins. The left panel shows proteins from *Chroococcidiopsis cubana* cells (CCALA 043) extracted using ACN/2% (v/v) TFA, 7:3, v/v, and separated in a 10% polyacrylamide gel under denaturing conditions (right separation lane; Bio-Safe Coomassie staining). The protein marker in the left separation lane contained Precision Plus Protein All Blue Prestained Protein Standards (Bio-Rad); the molecular masses from the top correspond to 250, 150, 100, 75 (thicker band), 50 (thicker band), 37, 25 (thicker band), 20, 15 and 10 kDa. The right panel depicts how 14 fractions were selected and cut out for the subsequent in-gel protein digestion (fraction numbering is provided on the right).

The optimized matrix solvent [ACN/2% (v/v) TFA, 7:3, v/v] was further used to extract proteins from cyanobacterial cells under conditions mimicking the on-target extraction, which occurs during the sample deposition step. The idea was to obtain identification information, which would help to assign peaks from the characteristic profile spectra to specific proteins. The extracts were stored frozen at -20°C and, on freezing, they separated into two phases: the bottom phase was an ice block, the top phase remained liquid and contained concentrated green (or violet in the case of *Gloebacter violaceus*) pigments (Fig 3). Protein identifications were done for the cyanobacterial species *Chroococcidiopsis cubana* (CCALA 043), *Gloeobacter violaceus* (PCC 7421) and *Synechococcus* sp. (UPOC S4). To prepare protein samples for 1-D gel electrophoresis, only the bottom extract phase was used as it showed much higher protein content than the top organic phase and also to get rid of the pigments. Upon evaporation of the solvent, the amount of the residual solid material appeared between 15–30 mg (from 200-ml cell cultures) and this was additionally extracted by Laemmli sample buffer. The subsequent separation of cyanobacterial proteins by SDS-PAGE is shown for CCALA 043 in Fig 4. After visualization, fractions of the separated proteins were subjected to in-gel digestion by modified trypsin. Peptides from the digests were desalted and analyzed by nanoLC coupled to ESI-MS/MS or MALDI-MS/MS followed by database searches. The searches were facilitated by the availability of genomic sequencing data for *Gloeobacter violaceus* (strain PCC7421; 4406 proteins) and *Synechococcus* sp. (40 different strains, 2000–3500 proteins for each strain) in the Swiss-Prot database. For the genus *Chroococcidiopsis*, there was only one proteome sequence available in the database (*Chroococcidiopsis thermalis*, strain PCC 7203; 5470 proteins).

The protein identification results are summarized in S1 Table. Among the identified proteins, there were typically photosynthesis-related proteins (proteins from photosystems I and II, cytochromes, plastocyanin, phycocyanins, phycoerythrins, phycobilisome linkers), ribosomal proteins, nucleic acids-related proteins, redox and transport proteins, enzymes, regulatory proteins as well as numerous proteins with yet uncharacterized function. Using nanoLC-ESI-MS/MS, 158 proteins with a calculated molecular mass below 20 kDa were identified for CCALA 043, 135 proteins for PCC 7421 and 167 proteins for UPOC S4. By means of nanoLC-MALDI-MS/MS, the corresponding numbers were 54, 40 and 67 proteins, respectively. From these, 36 proteins were assigned (based on an agreement in molecular mass) to the characteristic peptide peaks in the acquired IC MALDI-MS profile spectrum for CCALA 043 (34 from the ESI results, 17 from the MALDI results; 15 were found using both ionization techniques); Table 2. In the case of PCC 7421, 21 proteins matched (18 from the ESI results, 7 from the MALDI results; 4 were found using both ESI and MALDI). Finally, for UPOC S4, 24 proteins matched to the IC MALDI mass spectrum: 16 were from the ESI results, 9 from the MALDI results, and only a single protein was found from both ESI and MALDI experiments.

**Table 2:** Proteins assigned to the characteristic peaks in the profile IC MALDI mass spectrum of *Chroococcidiopsis cubana* (CCALA 043). Shadowing indicates which of these proteins were confirmed by both nanoLC-ESI-MS/MS and nanoLC-MALDI-MS/MS. The mass values are in Da, SC stands for sequence coverage.

Accession number	Protein	Monoisoto-pic Mass	Avg. Mass	Monoisoto-pic Mass (- Met)	Avg. Mass (-Met)	Corresp. Peak (*m/z*)	p*I*	ESI Score	Pepti-des	SC [%]
K9TYK3	Cytochrome b559 subunit beta	5059.7	5063.0	4928.6	4931.8	4933	10.8	231.1	5	100.0
K9TUG4	Photosystem one PsaX	5275.9	5279.2	5144.9	5148.0	5151	9.7	160.7	3	50.0
K9U763	Uncharacterized protein	5294.7	5298.1	5163.7	5166.9	5167	10.0	70.4	2	35.6
K9U1S7	CAB/ELIP/HLIP-like protein	6268.3	6272.4	6137.2	6141.2	6145	6.5	55.7	1	15.8
K9TXV0	CsbD family protein	6289.2	6292.9	6158.1	6161.7	6163	5.3	35.0	1	25.4
K9TWX6	50S ribosomal protein L32	6646.5	6650.6	6515.5	6519.4	6519	9.9	31.8	1	25.4
K9U0Q2	Uncharacterized protein	7009.7	7014.0	6878.6	6882.8	6879	4.4	143.5	3	61.7
K9U1N1	30S ribosomal protein S21	7160.1	7164.4	7029.0	7033.2	7034	11.9	147.0	3	54.2
K9U6A9	Uncharacterized protein	7958.2	7963.2	7827.1	7832.0	7837	4.8	72.5	2	35.1
K9TWD9	Molybdenum-pterin binding domain-containing protein	8181.1	8186.3	8050.1	8055.1	8057	8.9	47.0	1	22.9
K9U2Y0	Uncharacterized protein	8260.6	8265.9	8129.6	8134.7	8133	4.2	74.7	1	15.1
K9TYD6	30S ribosomal protein S18	8313.8	8318.9	8182.7	8187.7	8189	10.9	465.9	12	70.4
K9U6N7	Uncharacterized protein	8666.5	8672.0	8535.5	8540.8	8542	8.2	315.1	6	75.3
K9TTL0	50S ribosomal protein L29	8901.9	8907.3	8770.8	8776.1	8777	8.1	129.8	2	20.8
K9TX05	50S ribosomal protein L28	9132.9	9138.7	9001.8	9007.5	9008	10.9	44.3	1	11.5
K9TXI0	Uncharacterized protein	9621.8	9628.0	9490.8	9496.8	9492	6.1	47.7	1	14.5
K9U2M6	50S ribosomal protein L27	9987.3	9993.4	9856.3	9862.2	9864	11.3	297.6	7	61.1
K9TXE7	Glutaredoxin	10137.1	10143.6	10006.1	10012.4	10007	6.7	104.6	3	47.8
K9U5C3	Uncharacterized protein	10141.1	10147.4	10010.0	10016.2	10020	6.7	70.9	1	11.5
K9TSC9	30S ribosomal protein S19	10227.5	10233.9	10096.5	10102.7	10104	10.8	284.6	6	38.0
K9TYB6	30S ribosomal protein S15	10370.6	10376.9	10239.5	10245.7	10245	10.2	249.3	5	52.8
K9U6F7	Uncharacterized protein	10569.1	10576.0	10438.1	10444.8	10442	4.5	366.8	6	66.0
K9TTV4	30S ribosomal protein S20	11396.2	11403.3	11265.1	11272.1	11272	10.8	235.7	6	28.0
K9U7G6	Uncharacterized protein	11421.1	11428.3	11290.1	11297.1	11297	4.9	291.2	5	48.0
K9U809	Phosphoribosylformylglycinamidine synthase subunit PurS	11541.8	11549.3	11410.8	11418.1	11415	5.3	38.5	1	10.9
K9U2Y9	RNP-1 like RNA-binding protein	11601.7	11608.9	11470.7	11477.7	11477	6.4	359.8	7	52.4
K9TYP4	Uncharacterized protein	11780.0	11787.5	11649.0	11656.3	11654	5.3	300.0	6	36.4
K9U8M9	Glutaredoxin	11989.1	11996.8	11858.0	11865.6	11867	6.7	122.3	2	10.7
K9TWE1	30S ribosomal protein S6	12204.2	12211.9	12073.1	12080.7	12077	4.9	347.6	8	65.4
K9TYF8	Photosystem I reaction center subunit IV	13392.0	13400.1	13260.9	13268.9	13267	9.4	1210.2	18	75.8
K9U057	50S ribosomal protein L7/L12	13676.2	13684.9	13545.2	13553.7	13552	4.7	329.8	6	71.8
K9U7R5	Plastocyanin	14616.6	14626.0	14485.6	14494.8	14489	9.1	156.8	2	21.6
K9U6S7	Photosystem I protein PsaD	15766.2	15776.1	15635.1	15644.9	15640	9.5	1133.4	27	86.6
K9U8F2	Ribosome maturation factor RimP	17105.9	17116.5	16974.9	16985.3	16985	4.9	39.0	1	7.2
K9U3T4	Allophycocyanin beta subunit apoprotein	17703.1	17714.3	17572.0	17583.1	17584	5.4	415.9	9	65.4
K9TY88	Photosystem I reaction center protein PsaF subunit III	18399.6	18411.4	18268.6	18280.2	18281	7.8	550.8	11	51.5

**Fig 5:**
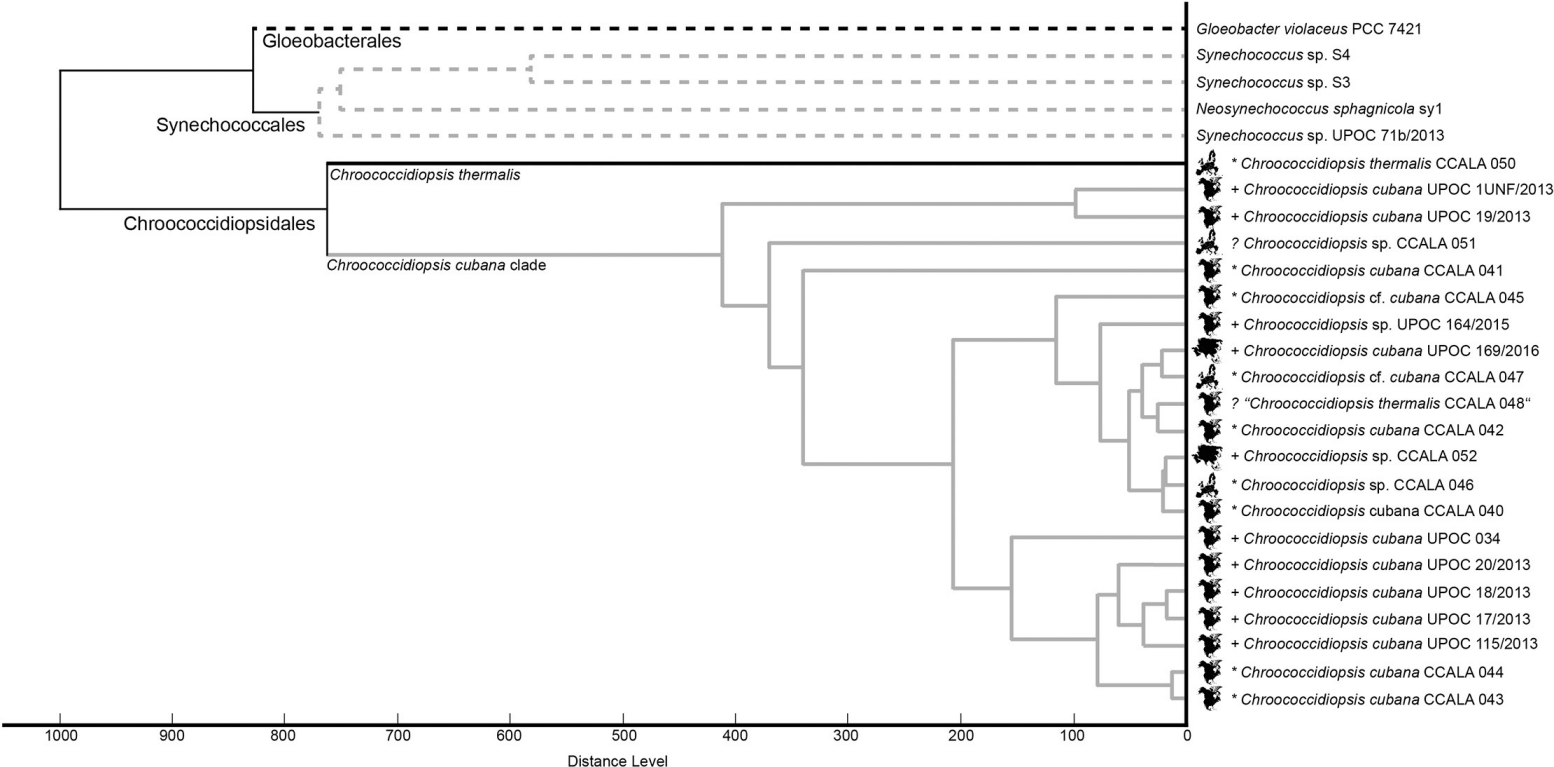
A similarity tree of *Chroococcidiopsis* species/isolates based on IC MALDI mass spectra. Multiple MALDI-TOF mass spectra acquired with intact cells of different *Chroococcidiopsis* species or various strains of the same *Chroococcidiopsis* species (distant taxa such as *Gloeobacter violaceus*, *Neosynechococcus sphagnicola* or *Synechococcus* sp. were also included into the group of the analyzed cyanobacteria) were processed in Biotyper 3.1 (Bruker Daltonik) to generate main spectral projections (MSPs). A library of MSPs was then used to construct the tree. The symbols at the *Chroococcidiopsis* species names refer to the habitat: asterisk = aquatic, plus = aerophytic, question mark = unknown. The black silhouettes of continents indicate the place of origin: Europe, North America or Asia. Based on our present results, the *Chroococcidiopsis thermalis* sample highlighted by quotation marks (CCALA 048) was probably misclassified in the collection.

**Fig 6:**
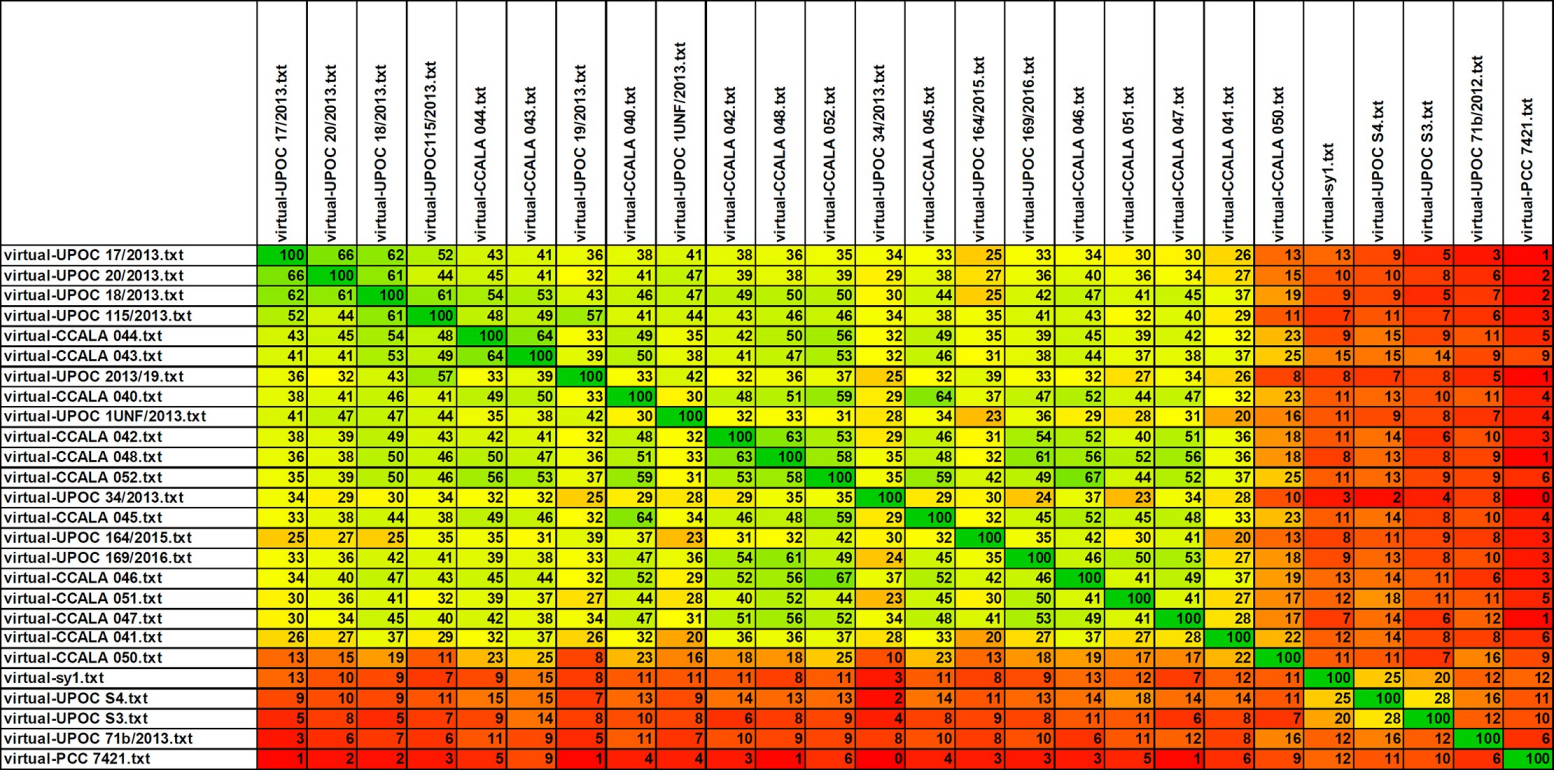
A map of similarities of *Chroococcidiopsis* species/isolates based on IC MALDI mass spectra. Multiple MALDI-TOF mass spectra acquired with intact cells of *Chroococcidiopsis* and other studied cyanobacteria were processed in Biospean [18] to generate virtual spectra (representing characteristic line spectra or “barcodes”) for individual samples. Then all virtual spectra were compared with each other to calculate percentage similarity values reflecting the presence of signals with the same *m/z* value (with a tolerance of ± 2 Da). The increase in the percentage value is displayed as a continuous color change in the order red (0) < dark orange < light orange < yellow (50) < light green < green < dark green (100).

**Fig 7:**
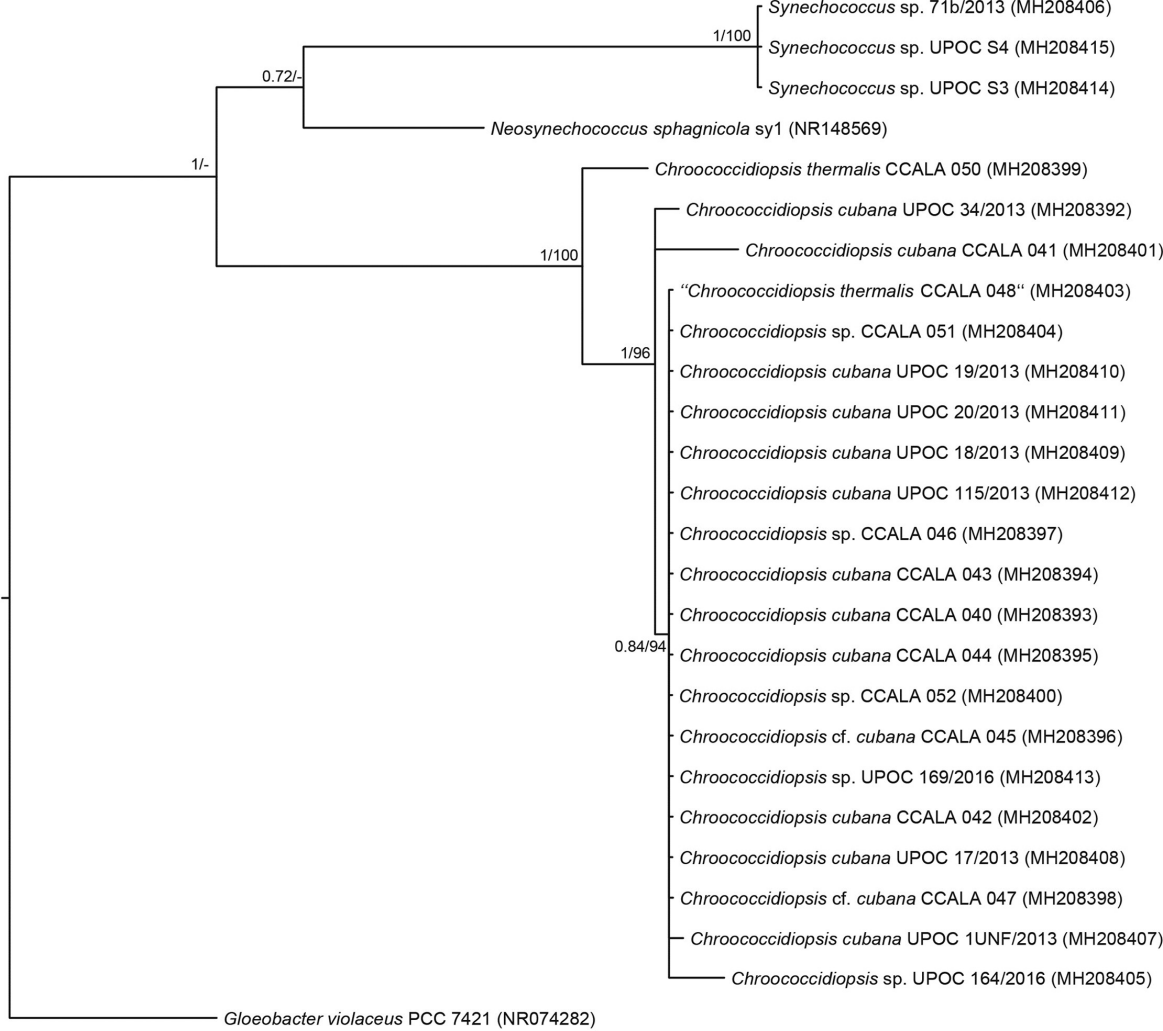
A phylogenetic tree based on sequencing of 16S rRNA gene. This consensus Bayesian tree includes sequences from 21 strains of the genus *Chroococcidiopsis*, 4 strains of *Synechococcus* sensu lato and *Gloeobacter violaceus* PCC 7421 as outgroup. Node supports are shown in the following order: Bayesian posterior probabilities and maximum likelihood. GenBank accession numbers of the respective 16S rRNA sequences are provided in parentheses.

The profile IC MALDI mass spectra of the investigated 26 cyanobacterial samples (each in 10 replicates acquired from 10 different sample spots on the target) were processed in the software Biotyper 3.1 (Bruker Daltonik) to generate main spectral projections (MSPs). These “averaged” line spectra were then used to construct a similarity tree (Fig 5). As can be seen, all *Chroococcidiopsis* isolates clustered together in several subgroups and only CCALA 050 appeared in a distance from the others. Another cluster comprised *Synechococcus* and *Neosynechococcus* species, *Gloeobacter violaceus* was a clear outgroup. Another tool, which was applied to evaluate the IC MALDI-MS results, Biospean, also demonstrated an overall similarity of the *Chlorococcidiopsis* isolates (Fig 6) displaying subgroup similarities (score values of around 50 in mutual comparisons) of the samples: 1) CCALA 043, CCALA 044; 2) UPOC 17/2013, UPOC 18/2013, UPOC 20/2013 and UPOC 115/2013; 3) CCALA 040, CCALA 042–048, CCALA 052, UPOC 169/2016. In another group, distant UPOC S3, UPOC S4 and sy1 clustered together. Interestingly, this kind of spectra processing also confirmed the distance of CCALA 050 (Fig 6). Phylogenetic analysis of 16S rRNA gene showed the presence of separate lineages containing *Gloeobacter violaceus* PCC7421, *Synechococcus* sensu lato and *Chroococcidiopsis*. The genus *Synechococcus* sensu lato was divided into separate lineages of *Neosynechococcus sphagnicola* and *Synechococcus* spp. The most distant clade belongs to the analyzed *Chroococcidiopsis* strains. Among them, *Ch*. *thermalis* strain CCALA 050 provided an individual lineage out of the *Chroococcidiopsis cubana* clade (Fig 7). The studied *Chroococcidiopsis* strains originated from aquatic and terrestrial habitats located in Europe, North America and Asia. Despite this fact they did not form separate branches reflecting their habitat or geographical location.

## Discussion

The genus *Chroococcidiopsis* (Chroococcidiopsidales, Cyanobacteria) contains primitive coccoid cyanobacteria with a high level of cryptic diversity and a high number of morpho- and ecotypes. The investigated group consisted of 21 strains obtained from scientific collections and assigned by phycologists at the species level (*Ch*. *cubana*, *Ch*. *thermalis*) or at least genus level (*Chroococcidiopsis* spp.). This was completed by a few well-known species from other taxonomical orders. *Gloeobacter violaceus* PCC 7421 (Gloeobacterales, Cyanobacteria) shows a simple morphology (e.g. missing thylakoids) and its molecular characteristics predetermine this evolutionary old strain for phylogenetic analyses (as an outgroup) or morphological comparisons. The four strains of *Synechococcus* sensu lato (Synechococcales, Cyanobacteria) represent a world-wide occurring genus sharing simple interspecific morphological similarity but exhibiting a high level of polyphyletism. The total set of 26 samples was subjected to comparative IC MALDI-MS and 16S rRNA analyses for classification purposes.

The use of MALDI-TOF MS for intact microorganisms is challenging. There is a demand for standardization of the whole procedure including cell culturing, sample preparation, instrument calibration and data analysis. Certain inconsistencies may appear because of differences in growth conditions, which influence microbial physiology. Also, identical clone isolates, cultured in the same way, may produce partly nonidentical mass spectra because of chemical and technical factors of the measurement, which induce *m/z* value shifts and variations in peak intensity [33]. On the other hand, abundant and constitutive molecules, such as ribosomal proteins, provide major diagnostic signals [12]. The aim was to develop a MALDI-based method for the classification of Chroococcidiopsidales (with a larger applicability to other taxonomical orders of cyanobacteria) and identify proteins, which could be suggested as spectral markers.

Since the advent of MALDI-MS in the 1980s, various matrix compounds have been used ensuring the applicability of this technique to samples containing representatives of the four major classes of biological molecules: peptides and proteins, saccharides, nucleic acids and lipids [34]. The cinnamic acid derivatives FA and SA proved to be advantageous matrix compounds for measurements with intact proteins [35,36]. In this work, the binary FA:SA mixture 5:15 mg·ml^-1^ was found optimal with respect to the number of the detected diagnostic protein ion species and their intensities. As a part of the optimization procedure, the influence of increasing TFA concentration in the matrix solvent was evaluated. For the analyzed cyanobacteria, 2% TFA was a good choice for acquiring rich peptide/protein profiles. In a previous IC MALDI-TOF study with *Saccharomyces cerevisiae*, 25% FoA was applied prior to the addition of matrix, which increased the number of peaks and their signal-to-noise-ratios in the mass spectrum [37]. But this strategy brought no further improvement when measuring with cyanobacteria.

It has long been known that the marker proteins producing characteristic signals in the profile MALDI-TOF mass spectra of whole cells of many bacterial or yeast species are ribosomal proteins [38]. Typically, ribosomal proteins are abundant and also basic, which favors their ionization. There are about 17,000 copies on average per a bacterial cell and this may represent an amount of about 3% of the total cellular mass [38]. But it is not possible to identify these proteins based on an agreement in molecular mass as more of them can be attributed to a single peak with a tolerance limit of 3 Da [39]. Fragmentation analyses performed directly with precursor ions selected during IC MALDI-MS are rare [40], obviously for two reasons: 1) the amount of peptide/protein compounds extracted on-target from the cells is low; 2) singly charged precursors with masses over 5000 Da (or even 10,000 Da) are not easily amenable to a fragmentation and extensive reading of the fragment masses as demonstrated by a study with ubiquitin and thioredoxin [41]. But there is the possibility to extract peptides and proteins from a large initial amount of microbial cells (30–100 mg) under conditions mimicking the on-target extraction process followed by their digestion and identification via reversed-phase liquid chromatography of peptides coupled to MS/MS analysis. Then the resulting sequence-based molecular mass data can be assigned to the *m/z* values acquired in IC MALDI-MS. We have recently applied this approach to plant pathogenic fungi [11].

We could certainly expect the presence of signals originating from ribosomal proteins in the profile MALDI-TOF mass spectra of the analyzed cyanobacteria. Additionally, as cyanobacteria belong to photosynthetizing organisms, many proteins related to photosynthesis are widely distributed inside the cell (e.g. in association with the thylakoid membrane) and abundant. Indeed, the identification experiments performed with extracts simulating the sample preparation step in IC MALDI-MS allowed to assign 30S and 50S ribosomal proteins (e.g. *m/z* 9831 and 7551 plus 10888, respectively, for UPOC S4) as well as photosystem I and II protein components, phycobilisome proteins, nucleic acids-binding proteins, nitrogen-metabolism and chemotaxis proteins, cytochromes plus other enzymes and various uncharacterized proteins to a significant part of the measured IC MALDI-MS signals for CCALA 043 (*Chroococcidiopsis cubana*), PCC 7421 (*Gloeobacter violaceus*) and UPOC S4 (*Synechococcus* sp.). This was facilitated by the availability of the genome sequences. Unfortunately, many intense signals could not be attributed to a particular protein (S1 Table). Phycobiliproteins, which covalently bind phycobilins as chromophores, were found frequent among the identification results from nanoLC-ESI MS/MS or nanoLC-MALDI MS/MS but their assigning to sequence-predicted molecular masses was not possible because of the unspecified quality and quantity of posttranslational modification. Interestingly, if intense peaks in the IC MALDI mass spectra could be assigned to the identified proteins for CCALA 043, then these mostly referred to yet uncharacterized proteins (e.g. *m/z* 6879, 7837, 8133, 9492 and 10020). As photoautotrophs, cyanobacteria cannot survive without photosynthesis. On the other hand, they can adapt themselves flexibly to light conditions by alterations in the representation of individual phycobiliproteins to reflect wavelength shifts in the utilizable light spectrum. Phycobiliproteins are abundant and constitute as much as 50% of the whole protein mass in a cyanobacterial cell [42]. The protein components of the photosystems I and II, photosynthetic protein electron carriers as well as phycobiliproteins can be considered permanent components of living cyanobacterial cells under standard environmental conditions. As such, they complement ribosomal proteins as typical markers of microorganisms in IC MALDI-MS.

The use of molecular markers such as 16S rRNA revealed the existence several main phylogenetic lineages of cyanobacteria including the orders Gloeobacterales, Synechococcales, Chroococcales, Chroococcidiopsidales, Spirulinales, Pleurocapsales, Oscillatoriales and Nostocales [5]. In addition to 16S rRNA, additional markers (e.g. ITS region, *nifH* or *cpcA*-*cpcB* genes) were successfully used in the classification of cyanobacteria [23, 43–45]. The topologies of the similarity trees constructed in this work correspond to previous data [46, 47]. Surprisingly, there are previous reports indicating that *Chroococcidiopsis thermalis* and *Ch*. *cubana* did not form separate lineages [48]. It follows that the species concept within the genus *Chroococcidiopsis* is based on delicate details which can easily be overlooked or undervalued. Thus, it is highly probable that misidentified strains and sequences exist as well as a high number of strains, which are identified only on the generic level. It is therefore highly desirable to verify strains prior to their use in phylogenetic and taxonomic studies. These circumstances also negatively influence the understanding of intra- and interspecific variability of the genus *Chroococcidiopsis* and MALDI-TOF MS is at hand to make it clear as it has been established as a fast and sensitive method in the identification and classification of microorganisms [8, 49]. Only a few studies targeted to the identification of cyanobacteria by MALDI-TOF MS were performed using the genera *Microcystis* and *Anabaena*. The authors have shown a high potential of the method in the classification cyanobacteria with respect to sensitivity, short operation time and low cost. Sun et al. identified two non-toxic and three toxic clades of *Microcystis aeruginosa* using MALDI-TOF MS ribosomal protein profiling [15]. Inside each clade, they found a quite long distance among strains indicating their high variability and sensitivity of MALDI-TOF MS method on strain level. Another analysis of mass spectra obtained from 21 cyanobacterial strains showed a clear intergeneric delimitation between *Anabaena* spp. and *Microcystis aeruginosa* [16].

In the phylogenetic trees constructed from IC MALDI-MS results and DNA sequencing (Figs 5 and 7, respectively), the genus *Chroococcidiopsis* forms a coherent clade split into two branches. *Ch*. *thermalis* CCALA 050 appears in a branch separated from the others, which is in agreement with its different morphological and molecular features as well as ecology (S1 Fig; Table 1). The distant position of CCALA 050 was obvious also from the Biospean data processing (similarity with the *Ch*. *cubana* clade was only 8–25%; Fig 6). The second branch includes *Ch*. *cubana* strains or species previously identified only at the genus level. The strain CCALA 048, designated as *Ch*. *thermalis*, originates from Cuba but there is no detailed information available about its habitat. Geitler described *Ch*. *thermalis* from a thermal spring in Kadjaj, Sumatra, in 1934 [50]. Similarly, according to the AlgaeBase (National University of Ireland, http://www.algaebase.org), *Ch*. *thermalis* is a freshwater species occurring in thermal springs. A high variability of morphological features, polyphyletic character or low habitat preference indicates the existence of cryptic species or unclear species concept [6]. In this study, CCALA 048 appeared in the *Ch*. *cubana* clade, which is not unexpected as it is more similar to *Ch*. *cubana* CCALA 044 than to *Ch*. *thermalis* CCALA 050 (compare in S1 Fig, panels H, Q, Z, and in Fig 6). Now it seems clear that the strain CCALA 048 has been misidentified and should rather be designated as *Chroococcidiopsis cubana*. The species concept of *Ch*. *thermalis* should follow original Geitler’s concept. Records of *Ch*. *thermalis* from other than thermal habitats should be revised.

## Conclusions

To conclude, previous MALDI-based studies of cyanobacteria [15, 16] did not pay attention to the comparison of MALDI-TOF MS and 16S rRNA phylogeny in the classification of cyanobacteria. Yet, the whole current taxonomic concept has been made using 16S rRNA phylogeny [5]. In this work, we found using optimized experimental procedures that the topologies of the MALDI-based hierarchical tree and 16S rRNA phylogenetic tree were almost the same. A difference appeared in the case of *Neosynechococcus sphagnicola*. Based on IC MALDI-MS features (confirmed by both Biotyper and Biospean data processing), this cyanobacterium was found more similar to *Synechococcus* S3 and S4 than to *Synechococcus* sp. UPOC 71b/2013, whereas the 16S rRNA sequence analysis showed its even distance from all analyzed *Synechococcus* samples. A verification of data by DNA-based analysis is always desirable but it is worth of mentioning that these two strategies cannot provide absolutely the same results because of the static character of genome information versus dynamic proteome changes [51]. Despite the low number of fundamental studies, MALDI-TOF MS definitely represents a very fast and effective method for the classification of cyanobacteria. In this context, it has a big potential for physiological and ecological studies, biotechnology or applied phycology of this group of microorganisms.

## Supporting information

S1 Fig Morphological variability of the analyzed chroococcalean cyanobacteria. (A-C) *Gloeobacter violaceus* PCC 7421; (D) *Synechococcus* sp. UPOC S3; (E) *Synechococcus* sp. UPOC S4; (F) *Neosynechococcus sphagnicola* sy1; (G) *Synechococcus* sp. UPOC 71b/2013; (H) *Chroococcidiopsis thermalis* CCALA 050; (I, J) *Chroococcidiopsis cubana* UPOC 1UNF/2013; (K) *Chroococcidiopsis* sp. CCALA 051; (L) *Chroococcidiopsis cubana* CCALA 041; (M) *Chroococcidiopsis* cf. cubana CCALA 045; (N) *Chroococcidiopsis* sp. UPOC 164/2015; (O) *Chroococcidiopsis* sp. UPOC 169/2016; (P) *Chroococcidiopsis* cf. *cubana* CCALA 047; (Q) *Chroococcidiopsis thermalis* CCALA 048; (R) *Chroococcidiopsis cubana* CCALA 042; (S) *Chroococcidiopsis* sp. CCALA 052; (T) *Chroococcidiopsis* sp. CCALA 046; (U) *Chroococcidiopsis cubana* CCALA 040; (V) *Chroococcidiopsis cubana* CCALA 043; (W) *Chroococcidiopsis cubana* UPOC 18/2013; (X) *Chroococcidiopsis cubana* UPOC 17/2013; (Y) *Chroococcidiopsis cubana* UPOC 115/2013; (Z) *Chroococcidiopsis cubana* CCALA 044.
(PDF)

S2 Fig MALDI-TOF MS of intact cyanobacterial cells. First part: profile mass spectra were acquired for closely related *Chroococcidiopsis cubana* strains (CCALA 043 and CCALA 044) using the dried-droplet technique and FA:SA binary matrix. Second part: Profile mass spectra acquired for *Chroococcidiopsis thermalis* (CCALA 050; top) and *Chroococcidiopsis cubana* (CCALA 040; bottom) using the dried-droplet technique and FA:SA binary matrix.
(PDF)

S1 Table Protein identifications from cyanobacterial cells. All table sections summarize protein identifications achieved by LC-ESI-MS/MS and LC-MALDI-MS/MS. Proteins with molecular masses below 20 kDa are highlighted by a light orange background. Parameters of the identification by tandem mass spectrometry for each protein include the probability-based score, number of experimentally confirmed peptides, sequence coverage (SC) value in percents, and root mean square error (RMS90) in ppm. For each protein, the respective accession number in the Swiss-Prot database is provided together with the name (if available). Then molecular masses are shown, monoisotopic and average (in Da), calculated from the sequence using Protein Cutter (https://software.cr-hana.upol.cz/proteincutter/index.php). Both masses are also provided in such a form anticipating a cleavage of the N-terminal methionine. Isoelectric point is another parameter, which is calculated from the sequence.
(XLSX)
