# Supplementary material for: Intact cell MALDI-TOF mass spectrometric analysis of Chroococcidiopsis cyanobacteria for classification purposes and identification of possible marker proteins
Source: PLoS One. 2018 Nov 29;13(11):e0208275. doi: 10.1371/journal.pone.0208275 (PMC6264847; doi:10.1371/journal.pone.0208275)
Supplement: S1 Fig — (A-C) Gloeobacter violaceus PCC 7421; (D) Synechococcus sp. UPOC S3; (E) Synechococcus sp. UPOC S4; (F) Neosynechococcus sphagnicola sy1; (G) Synechococcus sp. UPOC 71b/2013; (H) Chroococcidiopsis thermalis CCALA 050; (I, J) Chroococcidiopsis cubana UPOC 1UNF/2013; (K) Chroococcidiopsis sp. CCALA 051; (L) Chroococcidiopsis cubana CCALA 041; (M) Chroococcidiopsis cf. cubana CCALA 045; (N) Chroococcidiopsis sp. UPOC 164/2015; (O) Chroococcidiopsis sp. UPOC 169/2016; (P) Chroococcidiopsis cf. cubana CCALA 047; (Q) Chroococcidiopsis thermalis CCALA 048; (R) Chroococcidiopsis cubana CCALA 042; (S) Chroococcidiopsis sp. CCALA 052; (T) Chroococcidiopsis sp. CCALA 046; (U) Chroococcidiopsis cubana CCALA 040; (V) Chroococcidiopsis cubana CCALA 043; (W) Chroococcidiopsis cubana UPOC 18/2013; (X) Chroococcidiopsis cubana UPOC 17/2013; (Y) Chroococcidiopsis cubana UPOC 115/2013; (Z) Chroococcidiopsis cubana CCALA 044. (PDF) [file pone.0208275.s001.pdf]

Šebela et al., Supplementary figure 1. Morphological variability of chroococcalean cyanobacteria analyzed using MALDI-TOF MS: (A-C) *Gloeobacter violaceus* PCC 7421; (D) *Synechococcus* sp. UPOC S3; (E) *Synechococcus* sp. UPOC S4; (F) *Neosynechococcus sphagnicola* sy1; (G) *Synechococcus* sp. UPOC 71b/2013; (H) *Chroococcidiopsis thermalis* CCALA 050; (I, J) *Chroococcidiopsis cubana* UPOC 1UNF/2013; (K) *Chroococcidiopsis* sp. CCALA 051; (L) *Chroococcidiopsis cubana* CCALA 041; (M) *Chroococcidiopsis* cf. cubana CCALA 045; (N) *Chroococcidiopsis* sp. UPOC 164/2015; (O) *Chroococcidiopsis* sp. UPOC 169/2016; (P) *Chroococcidiopsis* cf. *cubana* CCALA 047; (Q) *Chroococcidiopsis thermalis* CCALA 048; (R) *Chroococcidiopsis cubana* CCALA 042; (S) *Chroococcidiopsis* sp. CCALA 052; (T) *Chroococcidiopsis* sp. CCALA 046; (U) *Chroococcidiopsis cubana* CCALA 040; (V) *Chroococcidiopsis cubana* CCALA 043; (W) *Chroococcidiopsis cubana* UPOC 18/2013; (X) *Chroococcidiopsis cubana* UPOC 17/2013; (Y) *Chroococcidiopsis cubana* UPOC 115/2013; (Z) *Chroococcidiopsis cubana* CCALA 044.

Photographs follow on the second page.

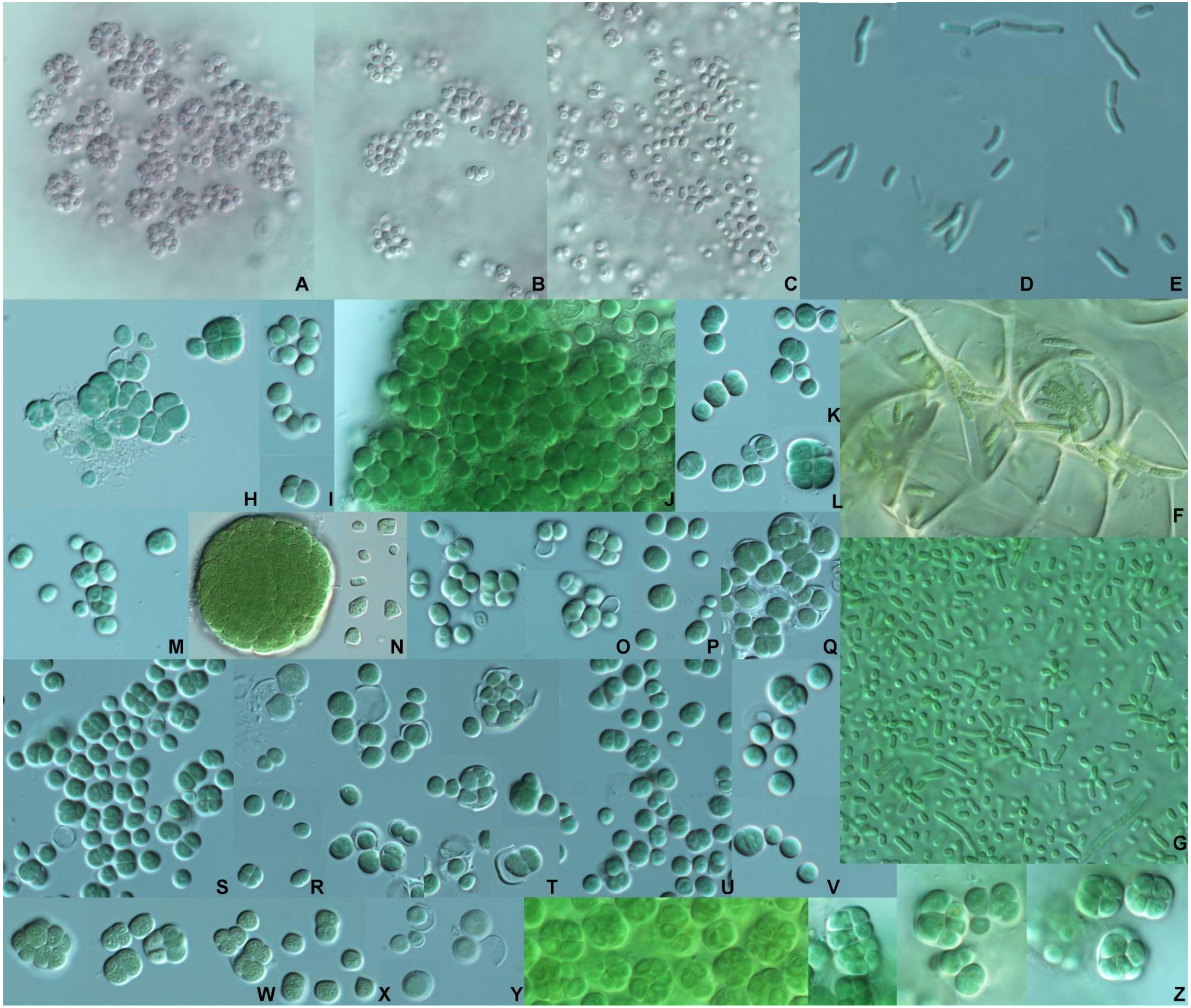
A
B
C
D
E
H
I
J
K
L
F
M
N
O
P
Q
G
S
R
T
U
V
W
X
Y
Z
