# Supplementary material for: Intact cell MALDI-TOF mass spectrometric analysis of Chroococcidiopsis cyanobacteria for classification purposes and identification of possible marker proteins
Source: PLoS One. 2018 Nov 29;13(11):e0208275. doi: 10.1371/journal.pone.0208275 (PMC6264847; doi:10.1371/journal.pone.0208275)

**Šebela et al., Supplementary figure 2.**

**MALDI-TOF MS of intact cyanobacterial cells.** Profile mass spectra acquired for closely related *Chroococcidiopsis cubana* isolates (CCALA 043 and CCALA 044) using the dried-droplet technique and formic acid-sinapinic acid binary matrix.

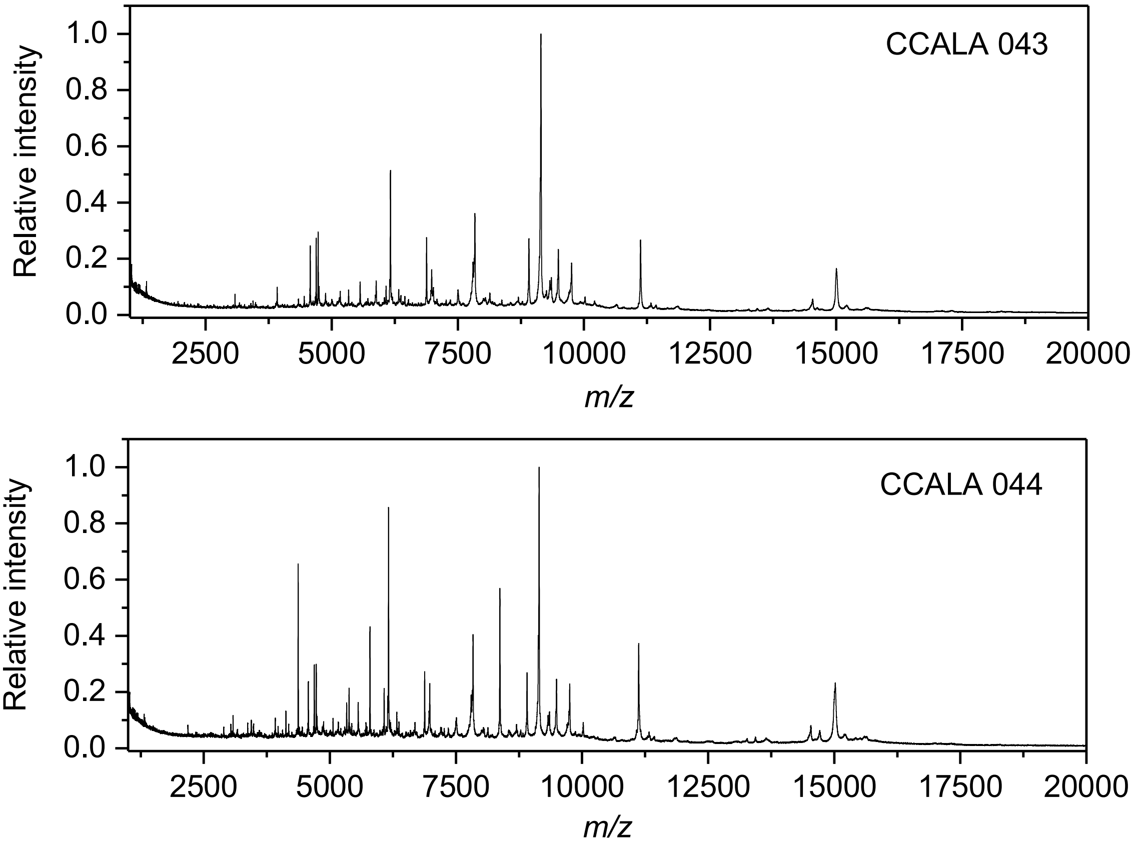

**MALDI-TOF MS of intact cyanobacterial cells.** Profile mass spectra acquired for *Chroococcidiopsis thermalis* (CCALA 050; top) and *Chroococcidiopsis cubana* (CCALA 040; bottom) using the dried-droplet technique and formic acid-sinapinic acid binary matrix.

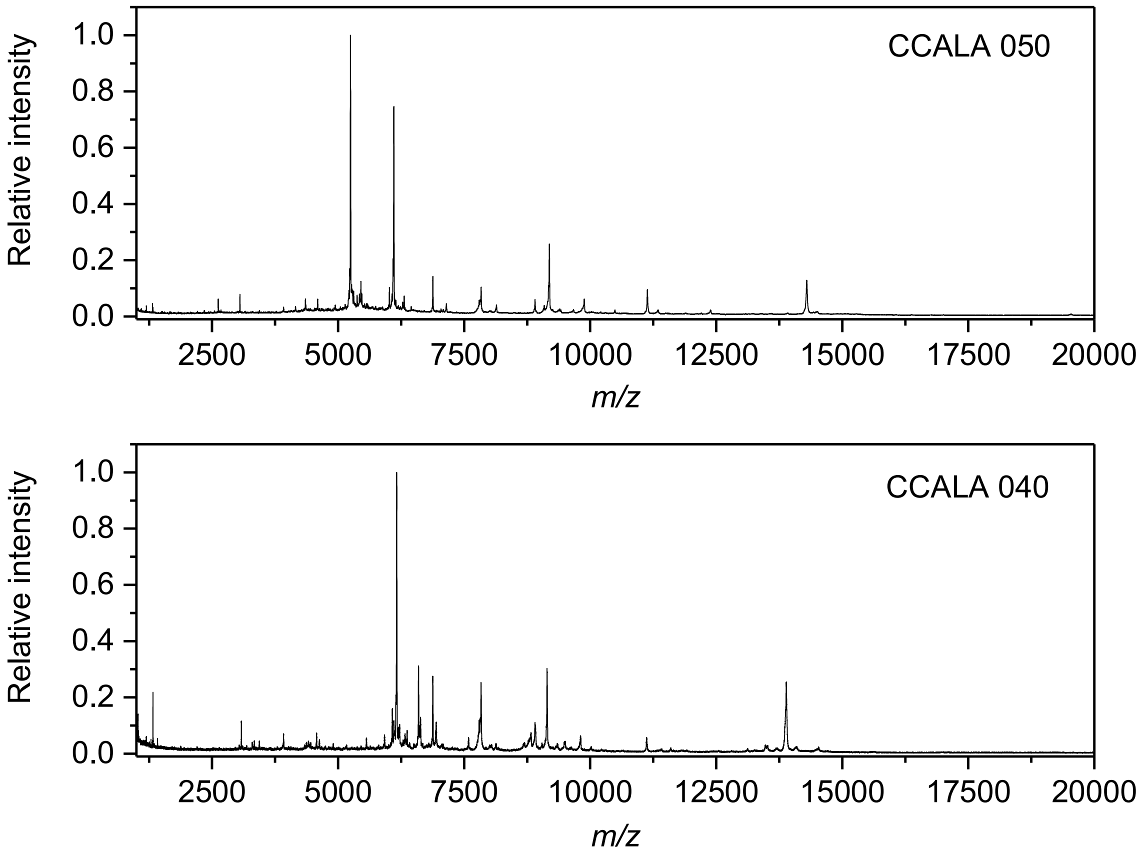

Supplement: S2 Fig — First part: profile mass spectra were acquired for closely related Chroococcidiopsis cubana strains (CCALA 043 and CCALA 044) using the dried-droplet technique and FA:SA binary matrix. Second part: Profile mass spectra acquired for Chroococcidiopsis thermalis (CCALA 050; top) and Chroococcidiopsis cubana (CCALA 040; bottom) using the dried-droplet technique and FA:SA binary matrix. (PDF) [file pone.0208275.s002.pdf]
